# [Retracted] miR‑593 inhibits proliferation and invasion and promotes apoptosis in non‑small cell lung cancer cells by targeting SLUG‑associated signaling pathways

**DOI:** 10.3892/mmr.2025.13490

**Published:** 2025-03-10

**Authors:** Fang Wei, Mofei Wang, Zhen Li, Yong Wang, Yong Zhou

Mol Med Rep 20: 5172–5182, 2019; DOI: 10.3892/mmr.2019.10776

Following the publication of this paper, and subsequently to the publication of a corrigendum (DOI: 10.3892/mmr.2021.12555) that was intended to address the issue of a pair of duplicated data panels in Fig. 5D, it was drawn to the Editor's attention by a concerned reader that certain of the flow cytometric plots shown in Fig. 4A and B and cell migration assay data shown in the originally published version of Fig. 5A were strikingly similar to data that had appeared previously in other papers written by different authors at different research institutes.

In view of the fact that the abovementioned data had already apparently been published prior to its submission to *Molecular Medicine Reports*, the Editor has decided that this paper should now be retracted from the Journal. The authors were asked for an explanation to account for these concerns, but the Editorial Office did not receive a reply. The Editor apologizes to the readership for any inconvenience caused.

